# *Brucella abortus* S19 GFP-tagged vaccine allows the serological identification of vaccinated cattle

**DOI:** 10.1371/journal.pone.0260288

**Published:** 2021-11-22

**Authors:** Carlos Chacón-Díaz, Ana Zabalza-Baranguá, Beatriz San Román, José-María Blasco, Maite Iriarte, Dariana Salas-Alfaro, Gabriela Hernández-Mora, Elías Barquero-Calvo, Caterina Guzmán-Verri, Esteban Chaves-Olarte, María-Jesús Grilló, Edgardo Moreno

**Affiliations:** 1 Centro de Investigación en Enfermedades Tropicales, Facultad de Microbiología, Universidad de Costa Rica, San Pedro, San José, Costa Rica; 2 Instituto de Agrobiotecnología, CSIC-Gobierno de Navarra, Mutilva, Navarra, Spain; 3 Unidad de Sanidad Animal, Centro de Investigación y Tecnología Agroalimentaria (CITA), Gobierno de Aragón, Aragón, Zaragoza, Spain; 4 Departamento de Microbiología y Parasitología, Instituto de Salud Tropical, Universidad de Navarra, Pamplona, Navarra, Spain; 5 Servicio Nacional de Salud Animal, Ministerio de Agricultura y Ganadería, Lagunilla, Heredia, Costa Rica; 6 Programa de Investigación en Enfermedades Tropicales (PIET), Escuela de Medicina Veterinaria, Universidad Nacional, Lagunilla, Heredia, Costa Rica; East Carolina University Brody School of Medicine, UNITED STATES

## Abstract

Bovine brucellosis induces abortion in cows, produces important economic losses, and causes a widely distributed zoonosis. Its eradication was achieved in several countries after sustained vaccination with the live attenuated *Brucella abortus* S19 vaccine, in combination with the slaughtering of serologically positive animals. S19 induces antibodies against the smooth lipopolysaccharide (S-LPS), making difficult the differentiation of infected from vaccinated bovines. We developed an S19 strain constitutively expressing the green fluorescent protein (S19-GFP) coded in chromosome II. The S19-GFP displays similar biological characteristics and immunogenic and protective efficacies in mice to the parental S19 strain. S19-GFP can be distinguished from S19 and *B*. *abortus* field strains by fluorescence and multiplex PCR. Twenty-five heifers were vaccinated withS19-GFP (5×10^9^ CFU) by the subcutaneous or conjunctival routes and some boosted with GFP seven weeks thereafter. Immunized animals were followed up for over three years and tested for anti-S-LPS antibodies by both the Rose Bengal test and a competitive ELISA. Anti-GFP antibodies were detected by an indirect ELISA and Western blotting. In most cases, anti-S-LPS antibodies preceded for several weeks those against GFP. The anti-GFP antibody response was higher in the GFP boosted than in the non-boosted animals. In all cases, the anti-GFP antibodies persisted longer, or at least as long, as those against S-LPS. The drawbacks and potential advantages of using the S19-GFP vaccine for identifying vaccinated animals in infected environments are discussed.

## Introduction

Brucellosis, caused by species of the genus *Brucella* is a widespread disease that affects a great variety of domestic and wildlife hosts, including humans [1]. Bovine brucellosis is caused mainly by *B*. *abortus*, albeit *B*. *melitensis* may be the etiological agent when cattle cohabits with infected small ruminants [1]. The disease is zoonotic and responsible for important economic losses due to abortions and infertility, as well as restrictions in the marketing of dairy products and livestock trade. As with other zoonotic diseases, human brucellosis is prevented by controlling and eradicating the disease from the host species [2, 3] through a combined vaccination and testing and slaughtering the seropositive animals [4, 5].

*B*. *abortus* S19 (S19) is a smooth live attenuated vaccine developed over eighty years ago [6] and proven highly effective in protecting cattle against brucellosis [7]. Indeed, vaccination of cattle with S19 combined with adequate diagnostic testing and culling of the seropositive animals (known as “test and slaughter” programme), has been instrumental to eradicate bovine brucellosis from many countries [1, 3, 7–12]. Despite its success, S19 induces residual antibodies against the *Brucella* N-formyl perosamine homopolysaccharides that built the O-chain of the smooth lipopolysaccharide (S-LPS) and native hapten (NH) polysaccharides, the main antigens used in the diagnosis of brucellosis. This can cause positive reactions in serological tests that use these polysaccharides as antigens [3, 13, 14]. Consequently, these seropositive but healthy bovines are culled unnecessarily as part of the eradication programmes. Despite the existence of vaccination strategies and diagnostic tests capable to differentiate infected from S19 vaccinated animals [10–12, 14–16], the S19 vaccine was banned in the United States [17] and its application abandoned in many other countries [3, 18].

To decrease the anti-S-LPS and NH antibodies in S19 vaccinated animals several strategies can be followed. A practical strategy consists in using reduced doses of S19 (i.e. 5×10^9^ CFU) and limiting vaccination to young (3–5 months old) replacement heifers exclusively [10, 11, 19]. However, the best procedure to minimize the untoward S19 induced antibodies is the combination of conjunctival vaccination [3, 10, 11, 20] and the further testing with NH serological assays [14–16]. Although these strategies have diminished significantly the problem, a low proportion of S19 vaccinated cows (particularly when S19 is used in adult cattle) can develop persistent anti-S-LPS and NH antibodies, causing diagnostic interferences in eradication programmes [15, 19, 21].

To avoid the above diagnostic interferences some research groups have developed rough *Brucella* vaccine candidates devoid of O-chain and NH-polysaccharides, an idea pursued along different periods [22–28]. However, none of these rough vaccines have been proven to be simultaneously safe, free of diagnostic problems, and effective against bovine brucellosis [3, 29, 30]. Other approaches have been the generation of *Brucella* deletion mutants in immunogenic proteins that could be used as negative antigenic markers for differentiating vaccinated from infected animals [31–33]. Although some of these protein-deficient vaccines have similar efficacy as the corresponding parental strains, the associated diagnostic tests are not straightforward [31, 32, 34, 35].

A different strategy is the incorporation of xenogenic markers in the classical live attenuated *Brucella* vaccine strains that could allow the development of associated diagnostic tests capable of identifying vaccinated animals in infected contexts. In a murine model we proved that the S19 vaccine expressing the green fluorescent protein (GFP) can be a suitable candidate for inducing antibodies against GFP in S19-GFP immunized animals, allowing their further identification in GFP-associated diagnostic tests [36]. We have demonstrated also that *B*. *melitensis* Rev1::*gfp* vaccinated sheep induce antibodies against GFP, making this strategy a potential alternative for identifying Rev 1 vaccinated animals in *B*. *melitensis* infected environments [37].

In this work, we describe an S19 derivative strain (S19-GFP) containing the *gfp* gene integrated into chromosome II which constitutively expresses GFP. This S19-GFP strain keeps similar biological and immunological properties to the S19 reference vaccine strain and protects mice against experimental brucellosis. Moreover, the S19-GFP induces anti-GFP antibodies in vaccinated heifers, reinforcing the principle that *Brucella*-GFP vaccines may serve for the identification of vaccinated individuals.

## Material and methods

### Bacterial strains, growth conditions, and DNA extraction

*B*. *abortus* S19 was obtained from the culture collection of the Centro de Investigación y Tecnología Agroalimentaria (CITA) of Aragón, Spain. Virulent *B*. *abortus* 2308W and *B*. *abortus* 2308W-GFP strains were obtained from PIET collection, as described before [36, 38]. *E*. *coli* strains carrying specific plasmids for mini-*Tn7* based integration assays as reported elsewhere [39] were obtained from the culture collection of the Departamento de Microbiología, Universidad de Navarra, Pamplona, Spain. For GST-GFP expression, *E*. *coli* XL-1-blue carrying pGEX-GFP was grown as reported elsewhere [36]. DNA extracted from *E*. *coli* strain TOP10 (Invitrogen) was used as a negative control in some assays. Strains were stored at -80°C in skimmed milk (Scharlau). *Brucella* strains were routinely grown in Blood Agar Base N° 2 (BAB; Biolife), trypticase soy broth (TSB), and *E*. *coli* strains on Luria Broth (LB) either plain or supplemented with 100 μg/mL ampicillin, 35 μg/mL kanamycin, 5 μg/mL nalidixic acid, 15 μg/ml gentamicin, or 1 mg/mL erythritol, all from Sigma-Aldrich. Plasmid and chromosomal DNA were extracted with Qiaprep spin Miniprep (QIAGEN) and Ultraclean Microbial DNA Isolation Kit (Mo Bio Laboratories), respectively. Sigma-Genosys Ltd synthesized the primers. Genetic manipulation of *B*. *abortus* S19 for GFP tagging, as well as genetic confirmation of S19-GFP strain were performed at BSL-3 facilities at Universidad de Navarra, Spain (reference A/ES/05/I-09). S19 and S19-GFP vaccine preparation for *In vitro* and *In vivo* assays were performed according to the biosecurity conditions of the Centro de Investigación en Enfermedades Tropicales, and approved by the *Vicerrectoría de Investigación* of the University of Costa Rica (www.vinv.ucr.ac.cr), Costa Rica, protocols described in the *Red Temática de brucelosis*, 803-B3-761.

#### Construction and genetic characterization of the S19-GFP vaccine

The *Tn7* carrying the *gfpmut3* gene was inserted in chromosome II of *B*. *abortus* S19 by using the four-parental mating method previously described [37, 40].The expression of the *gfpmut3* gene is driven by the *E*. *coli rrnB* P1 ribosomal promoter, as described in [39]. The insertion site downstream of the conserved gene *glmS* and the orientation of the mini-*Tn7* were checked in the selected S19 clones by PCR using the following pairs of primers: (i) P_glmS___B_ (5´ GTCCTTATGGGAACGGACGT 3´) and P_Tn7-R_ (5´ CACAGCATAACTGGACTGATT 3´) detecting the upstream region of the mini-*Tn7* insertion; (ii) P_Tn7-L_ (5´ ATTAGCTTACGACGCTACACCC 3´) and P_recG_ (5´ TATATTCTGGCGAGCGATCC 3´) that detects the downstream region of the mini-*Tn7* insertion; and (iii) P_glmS_B_ and P_recG_ that amplifies the intergenic region in the absence of the mini-*Tn7*.

The presence of a unique copy of the mini-*Tn7* was determined by Southern-blot, using 1 μg of *Brucella* DNA digested with EcoRV (30 U) (New England Biolabs) at 37°C overnight. Digested DNA was resolved by agarose gel electrophoresis and transferred onto Hybond ™ N+ membrane (GE Healthcare) by capillarity action. A specific DNA probe was obtained by PCR using P_glmS_B2_ (5´ TCATCCTCATCACCGACAAG 3´) and P_Tn7-R_ (5´ CACAGCATAACTGGACTGATT 3´). The DNA fragments digested by EcoRV were detected by hybridization with horseradish peroxidase**-**labeled DNA probes, using the Amersham ECL direct nucleic acid labelling and detection system (GE Healthcare) according to the manufacturer’s instructions. After genetic confirmation, three positive clones were stored at -80°C, only one clone was selected for further analysis. The site of insertion was determined by carrying out a Sanger sequencing reaction using the primer P_glmS_B2_. The S19::*Tn7*-*gfp* vaccine construct is named *Brucella abortus* S19-GFP, or simply S19-GFP. The stability of the *gfp* gene insertion was assessed by PCR after 20 subcultures in BAB plates and after isolating the fluorescent S19-GFP from infected mice.

#### Phenotypic characterization of S19-GFP

The *B*. *abortus* S19-GFP strain was characterized following the standard *Brucella* typing procedures: colony morphology, crystal violet exclusion, catalase, oxidase, urease, and acriflavine agglutination tests, sensitivity to Tb, Wb, Iz, and R/C phages, agglutination with anti-A and anti-M monospecific sera, both CO_2_ and serum dependence, as well as susceptibility to thionine blue (20 μg/mL), fuchsine (20 μg/mL), safranin (100 μg/mL) and erythritol (1 mg/ml) [41]. Bacterial growth curves were determined by the number of bacterial colony-forming units (CFU) on BAB plates at selected time intervals from a 10 mL culture flask containing a suspension 1x10^3^ CFU/mL in TSB incubated at 37°C, at 200 rpm. GFP expression was evaluated in bacterial cultures under UV illumination and by fluorescence microscopy, as described previously [36]. The amount of GFP produced by the S19-GFP was estimated by Western blotting (WB) as described elsewhere [42]. The stability in the expression of GFP by S19-GFP was assessed by direct UV illumination after 20 subcultures in BAB plates and after two passages in mice.

#### Molecular identification of S19-GFP

A multiplex PCR-GFP was used for the differentiation of mini-*Tn7*-*gfp* tagged from untagged strains, as described previously [37]. The multiplex differentiates GFP tagged strains by the presence of a double amplicon band of 200 bp and 432 bp, corresponding to mini-*Tn7* insertion and *gfp* gene, respectively, or a 328 bp band in the absence of *Tn7-gfp* insertion in non-tagged *Brucella* strains.

#### Purification and stability of recombinant GFP

Recombinant GST-GFP was obtained by affinity chromatography as a glutathione-S-transferase (GST-GFP) fusion protein from the soluble fraction of *E*. *coli* XL1 Blue harboring plasmid pGEX-GFP. Endotoxicity of the purified GST-GFP was determined by Limulus lysate assay (Sigma-Aldrich), following product information technical bulletin. The stability and integrity of GST-GFP were tested as described previously [37].

### Cell culture assays

Cell infections for estimating bacterial invasion and replication were performed as described previously [43]. Briefly, HeLa cells were grown to sub-confluency in 24-well tissue culture plates. Infections were carried out using an overnight TSB culture of *B*. *abortus* S19-GFP, *B*. *abortus* S19, or *B*. *abortus* 2308-GFP diluted in Eagle´s minimal essential medium (Sigma-Aldrich) to reach the desired MOI 500. Plates were centrifuged at 1500 rpm for 15 minutes at 4°C, incubated for 1 hour at 37°C under 5% CO_2,_ and washed with a solution of 137 mM NaCl, 10 mM phosphate, 2.7 mM KCl, at a pH of 7.4 (PBS). Extracellular bacteria were killed by adding gentamicin (100 μg/mL) to the medium for 1 hour, and cells incubated then for the indicated times in the presence of 5 μg/mL gentamicin. Plates were washed with PBS, and cells lysed with 0.1% Triton X-100 for 10 min. Aliquots were plated in BAB and incubated at 37°C for three days for CFU assessment. Additionally, CFUs from *B*. *abortus* GFP tagged strains were exposed to UV light to confirm the fluorescent phenotype. Intracellular location of fluorescent bacteria was confirmed by two-fluorescent labelled protocols as described in [44].

### Residual virulence, protective efficacy, and serological studies in mice

CD-1 female mice were accommodated in the “Bioterio de la Universidad Nacional, Costa Rica.” All animals were kept in cages with water and food *ad libitum* under biosafety containment conditions. The animal handling and procedures were under the guidelines revised and approved by the ‘‘Comité Institucional para el Cuido y Uso de los Animales de la Universidad de Costa Rica” (CICUA 16–10) in agreement with the corresponding law ‘‘Ley de Bienestar de los Animales” of Costa Rica (Law 7451 on Animal Welfare).

For residual virulence studies, groups of 25 mice were intraperitoneally inoculated with 1×10^6^ CFU/mouse of S19-GFP or S19, and spleen counts assessed at various times after vaccination, as described previously [26]. After checking S19-GFP and S19 colonies isolated in the spleens of mice by fluorescence and PCR, the infection levels were expressed as the mean ± SD (n = 5) of the individual log_10_ CFU/spleen at each selected point time.

Standard procedures were followed for protective efficacy assessment [5, 45]. Briefly, groups of five mice each were immunized subcutaneously with 1×10^5^ CFU/mouse of S19-GFP or S19 strains. Additional groups (n = 5) of mice were inoculated with PBS and kept as unvaccinated controls. All mice were challenged intraperitoneally with 5×10^4^ CFU/mouse of virulent *B*. *abortus* 2308W at four weeks after vaccination. The log_10_ CFU/spleen of the virulent strain was determined in each mouse, two weeks after the challenge. Residual vaccine colonies were differentiated from those of challenging strain by double culture in BAB plates supplemented or not with erythritol as described elsewhere [26]. While the S19 and S19-GFP are inhibited by erythritol, *B*. *abortus* 2308W challenge strain growths in the presence of this sugar.

To determine the anti-GFP specific antibody response, groups of five mice were simultaneously inoculated with a mixture of 20 μg of GST-GFP plus 1x10^5^ CFU of S19 or with a mixture of 20 μg of GST-GFP plus 1x10^5^ CFU of S19-GFP. Then, mice were bled at different intervals, and the anti-GFP antibodies detected in serum samples by ELISA-GFP as described before [36].

### Cattle studies

Twenty-five eight to eleven months old female Brangus crossbred heifers were used for vaccination studies. Besides, 118 similar Brangus heifers from the same farm were kept as unvaccinated controls. All animals were born in the same brucellosis-free farm (San Carlos, Alajuela, Costa Rica) and placed in a brucellosis-free area for over 64 weeks. The bovines were handled according to regulation procedures that were approved by the ‘‘Comité Institucional para el Cuido y Uso de los Animales de la Universidad de Costa Rica” (CICUA 16–10) in agreement with the ‘‘Ley de Bienestar de los Animales”, Costa Rica (Law 7451 on Animal Welfare), and according to the “International Convention for the Protection of Animals” endorsed by Costa Rican Veterinary General Law on the National Service of Animal Health (Law 8495).

Previous to vaccination the 25 experimental and the 118 control heifers were tested with the Rose Bengal Test (RBT), competitive enzyme-linked immunoabsorbent assay (cELISA-S-LPS), and indirect enzyme-linked immunoabsorbent assay (iELISA-GFP) [5, 36] to confirm the absence of both anti-S/LPS and anti-GFP antibodies. Subsequently, the 25 experimental heifers were divided into four groups and vaccinated as described in Table 1.

**Table 1:** S19-GFP vaccination protocols of 25 crossbred heifers of 8–11 months of age.

Group	Vaccination route	N° of animals	Doses^a^	GST-GFP boost^b^	Week of boost
**A**	Subcutaneous^**c**^	5	S19-GFP	None	None
**B**	Subcutaneous	10	S19-GFP + GFP^**d**^	150 μg	7
**C**	Subcutaneous	5	S19-GFP	150 μg	7
**D**	Conjunctival^e^	5	S19-GFP	150 μg	7

^**a**^Individual S19-GFP vaccine suspensions contained 5×10^9^ CFU.

^**b**^Individual GST-GFP boosting doses were always diluted in 2 mL of a 1% sterile calcium alginate suspension and administered subcutaneously.

^**c**^Subcutaneous vaccine was prepared in 2 mL of 0.1M sterile PBS.

^**d**^When required the 300 μg of GFP given simultaneously with the S19-GFP was directly dissolved in the corresponding vaccine suspension.

^**e**^Conjunctival vaccine was prepared in 70 μL of 0.1M sterile PBS.

The S19-GFP inocula were prepared at 1x10^12^ CFU/mL, diluted in PBS pH 6.85 to the desired concentration, and retrospectively assessed, as detailed elsewhere [26]. Quality assurance of the inocula was assessed according to standard procedures [5]. The booster was performed with the indicated quantities (Table 1) of GST-GFP in 1% calcium alginate as adjuvant. All vaccinated animals and the unvaccinated heifers were bled regularly and tested serologically for up to three years.

Each serum sample was divided into 1 mL aliquots, frozen, and stored at -70°C until use.

### Serological procedures in cattle

An iELISA-GFP internal procedure for the detection of bovine anti-GFP antibodies was performed on polystyrene Immunolon II 96-well plates (Thermo Scientific) coated with 10 μg/mL GST-GFP antigen (100 μL/well) in 0.05 M carbonate buffer pH 9.6, for two hours at 37°C and then sealed and incubated overnight at 4°C. The plates were washed five times with PBS containing 0.1% (v/v) Tween 20 (PBS-T) (Sigma-Aldrich) to remove unbound antigen. The nonspecific sites were blocked by incubation with 100 μL of PBS-T containing 2% (w/v) skimmed milk powder per well for one hour at 37°C and then washed. Controls and samples were diluted 1:200 in PBS, and 100 μL of the corresponding dilution was added to each well in duplicate and incubated for one hour at 37°C in an orbital shaker. After another cycle of washing, 1:3000 diluted HRP-labelled Protein G (0.5 mg/mL stock, Thermo Scientific) in PBS containing 0.1% skimmed milk, was added to each well and incubated for one hour at room temperature. After incubation, the plates were washed in PBS-T, as described above. One hundred μL of ABTS substrate chromogen (Sigma-Aldrich) was added per well and incubated for 30 minutes in the dark at room temperature. The resultant green color reaction was stopped by adding 50 μl of 4% SDS per well, and the absorbance values were determined at 405 nm using an ELISA plate reader. Negative control serum, positive control serum, and dilution buffer were included in each plate.

For the validation of the iELISA-GFP diagnostic performance, we used as negative controls the sera from all experimental heifers (before vaccination) as well as additional control sera from 118 unvaccinated controls (brucellosis-free bovines) and 118 *Brucella* infected (culture positive) cattle (coming from different ages, breeds, physiological conditions, and epidemiological status, and obtained from the PIET sera bank collection, Veterinary Medicine School of the National University, Costa Rica [21]. As positive controls we used the 25 sera from the experimental heifers taken at 16 weeks after vaccination. A hyper-immune serum against GFP produced in a cow following immunization protocols described elsewhere [42] was used also as a positive control. All sera were run in duplicate and in three dilutions to determine the linearity of the reaction.

Moreover, for the detection of anti-GFP antibodies in sera from vaccinated heifers (1:500 dilution) a Western Blot (WB) was performed following previous protocols using purified GFP as antigen [46]. Serum from experimental heifers previous to vaccination as well as serum from brucellosis free bovines were used as negative controls.

Likewise, the RBT (performed according to standard procedures [5]) and a competitive ELISA (cELISA-S-LPS) [21] were used for the detection of anti-S/LPS antibodies in vaccinated animals. The c-ELISA cut-off value resulting in the optimal diagnostic performance was established as 30% positivity with the sera from the *Brucella* infected and brucellosis-free cows described above.

### Statistical analyses

Statistical comparison of means was performed by one-way ANOVA followed by Fisher’s Protected Least Significant Differences test.

## Results

### Verification of the *B*. *abortus* S19-GFP construction

**Fig 1:**
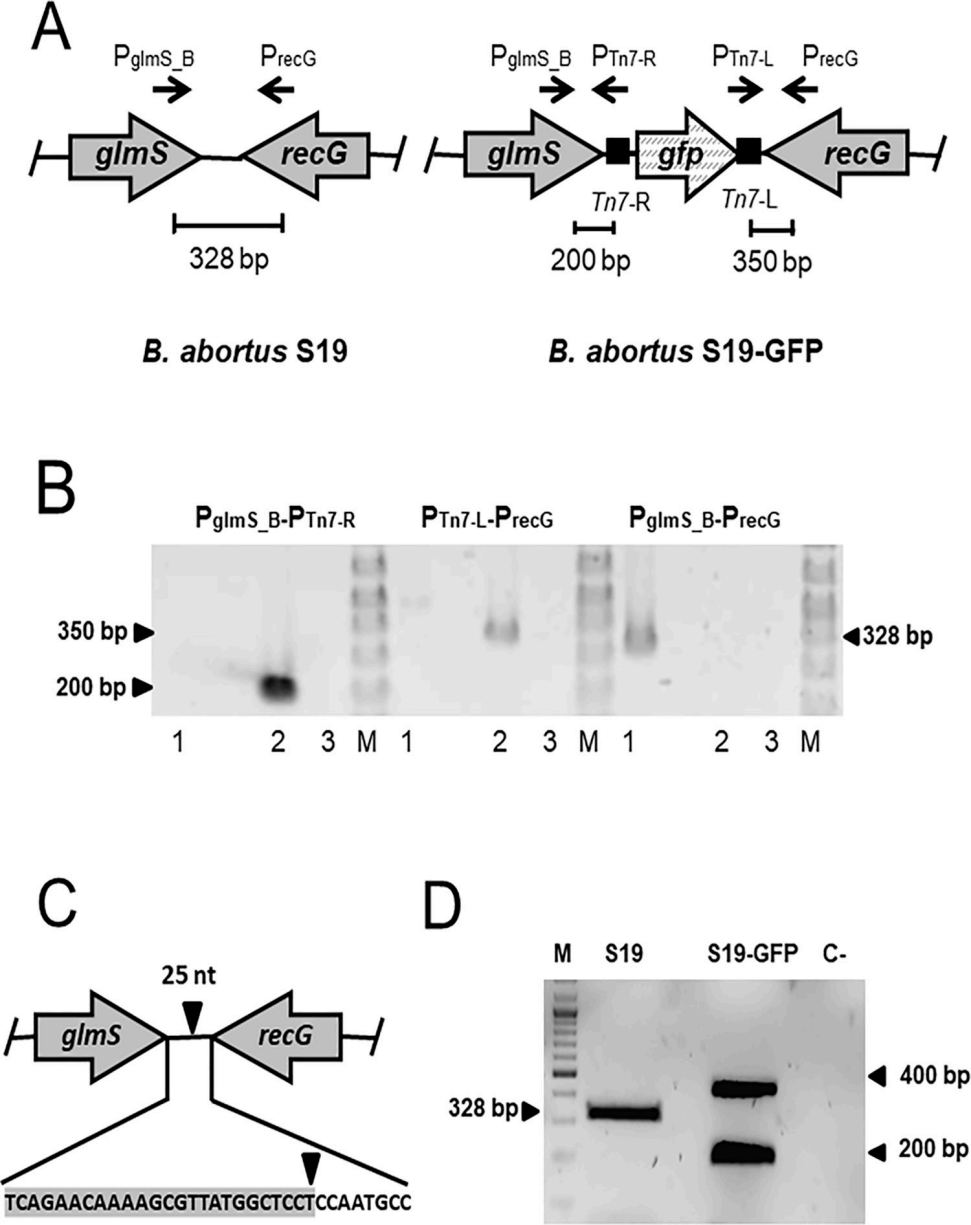
Construction of *B*. *abortus* S19-GFP vaccine. The integration of the *gfp* gene in *B*. *abortus* S19 was achieved using the mini-*Tn7* system through a four-parental mating strategy. (A) Schematic representation of the integration of mini-*Tn7* downstream of the *glmS* gene in chromosome II of *B*. *abortus* S19. (B) Verification of transposition was confirmed by PCR using primers pairs shown by convergent arrows that yield PCR fragments indicated in bp. Lane 1: DNA from *B*. *abortus* S19, lane 2, DNA from S19-GFP; lane 3, water. (C) The insertion site for the mini-*Tn7* in *B*. *abortus* S19 was determined to be in an intergenic region, 25 bp downstream of the *glm*S gene. (D) Differentiation of S19-GFP vaccine from reference *Brucella* strains by PCR. Mini-*Tn7*-GFP tagged vaccine amplified two bands (200 and 432 bp amplicons) that corresponded to the mini-*Tn7* and the *gfp* gene. In the untagged S19 strain, a unique 328 bp band of the intergenic region within *glmS* and *recG* genes is shown. “M”, molecular weight ladder; “C-“, negative DNA control from *E*. *coli* TOP10 strain.

We adapted the orientation-specific mini-*Tn7* to insert the *gfp* gene in chromosome II of *B*. *abortus* S19 downstream of the *glmS* gene (Fig 1A). Verification of transposition was established by PCR (Fig 1B), and Southern blot analysis confirmed the insertion of only one mini-*Tn7* carrying *gfp* gene per genome. As reported for other bacterial species [40], sequencing of the intergenic region downstream of *glm*S revealed that the insertion site is 25 nucleotides downstream of the *glm*S gene (Fig 1C). This S19-GFP strain is devoid of antibiotic-resistant cassettes and displays the same antibiotic sensitivity as the parental S19 strain. Two amplification bands corresponding to the S19-GFP tagged strain were indicative of the *gfp* gene, in contrast to a single band in the non-tagged S19 strain (Fig 1D).

### *B*. *abortus* S19-GFP displays similar biological properties to that of the S19 parental strain

**Fig 2:**
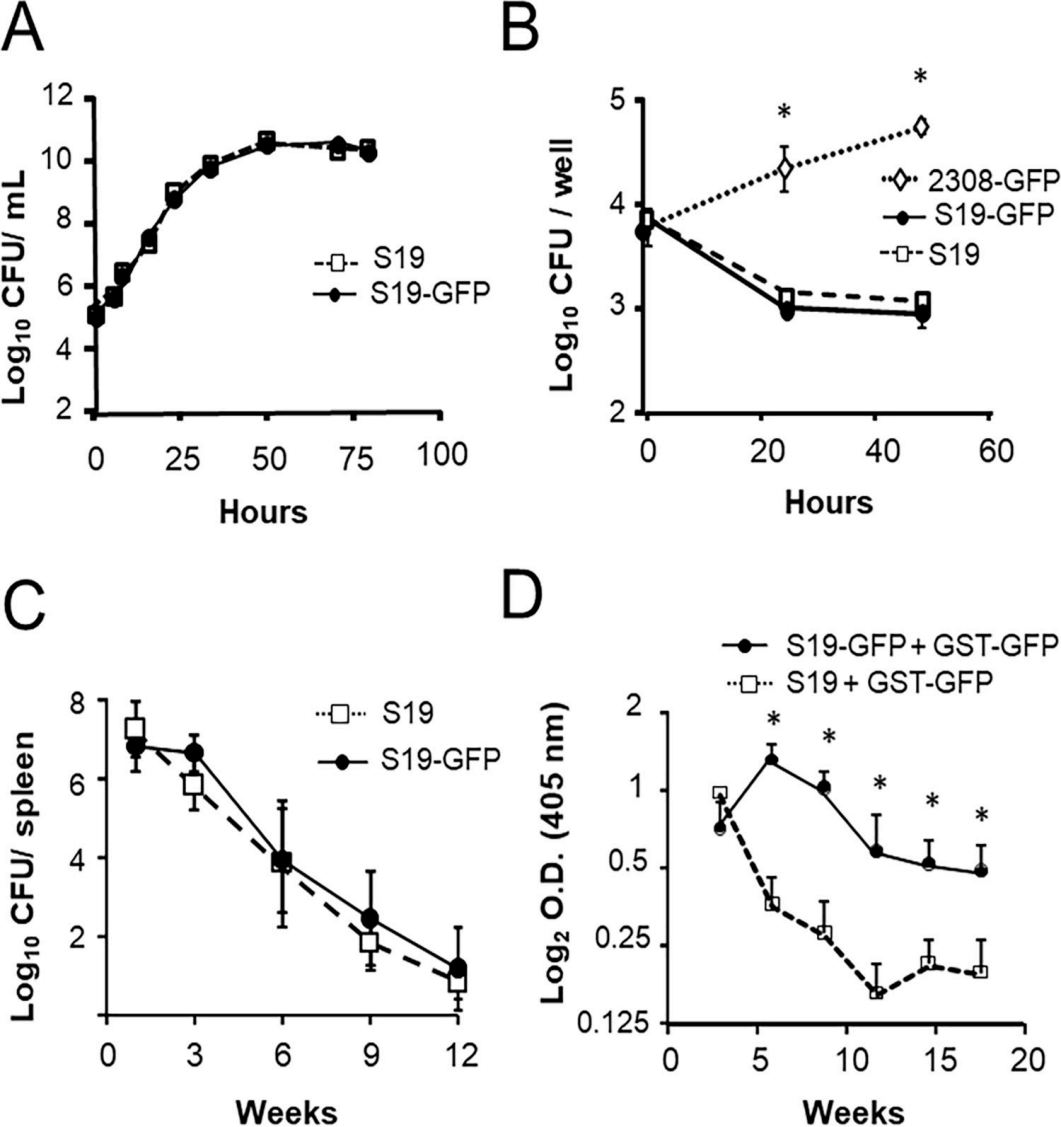
S19-GFP displays similar biological properties to the parental S19 strain and requires constitutive GFP expression for sustaining anti-GFP antibody levels in mice. (A) *In vitro* growth kinetics in TSB medium of *B*. *abortus* S19 and S19-GFP strains. (B) Replication kinetics of *B*. *abortus* S19-GFP, S19, and 2308-GFP strains in HeLa cells. (C) Groups of 25 mice were inoculated intraperitoneally with 1×10^6^ CFU of *B*. *abortus* S19-GFP or S19, and the mean ± SD (n = 5) of log_10_CFU/spleen were assessed at selected intervals. (D) Groups of 5 mice were intraperitoneally inoculated either with a mixture of 1×10^5^ CFU of S19 + 20 μg of GST-GFP or with a mixture 1×10^5^ CFU of S19-GFP + 20 μg of GST-GFP and the level of anti-GFP antibodies in serum (expressed as log_2_ OD) assessed by ELISA-GFP at the indicated times after inoculation. * Fisher´s PLSD test: p ≤ 0.05.

S19*-*GFP showed similar phenotypic and bacteriological characteristics, serological response and residual virulence and protection in mice to those of the corresponding isogenic parental strain (Fig 2 and Table 2). The kinetics of bacterial growth of S19*-*GFP was closely similar to that of S19 parental strain (Fig 2A). The S19-GFP invasion and replication profiles in HeLa cells were also similar to that of the parental S19 but were significantly different from those of the virulent *B*. *abortus* 2308-GFP strain (Fig 2B). Following two-fluorescent labeled protocols [44], we have determined that all the intracellular bacteria observable in HeLa cells were fluorescent. Likewise, all the GFP-tagged *B*. *abortus* CFUs recovered were fluorescent when exposed to UV light as previously reported [36, 37], confirming the constitutive expression of GFP.

**Table 2:** Protective efficacy of S19-GFP and S19 strains against virulent *B*. *abortus* in mice^a^.

Group	Log_10_ *B*. *abortus* 2308 CFU /spleen (mean ± SD)	Protection Units
**S19-GFP**	1.94 ± 1.08 ^b^	4.36
**S19**	2.52 ± 1.03 ^b^	3.78
**Unvaccinated**	6.30 ± 0.23	--

^**a**^ Groups of CD-1 mice (n = 5) were vaccinated subcutaneously with 1x10^5^ *B*. *abortus* S19-GFP CFU or 1x10^5^ *B*. *abortus* S19 CFU. The unvaccinated controls were injected subcutaneously with 0.1 mL of sterile PBS. Four weeks after that, all mice were challenged intraperitoneally with 5x10^4^ CFU of virulent *B*. *abortus* 2308 strain. Two weeks later, the number of viable bacteria in the spleen was determined. The efficacy of vaccination was expressed as the mean ± SD (n = 5) of individual log_10_ CFU/spleen of *B*. *abortus* 2308 challenge strain.

^**b**^ *p* < 0.001 *vs*. unvaccinated controls. No significant differences were obtained between S19 and S19-GFP vaccines.

The S19-GFP replication kinetics in the spleen of mice was similar to that obtained with the corresponding S19 parental strain (Fig 2C). As shown in Fig 2D, mice inoculated simultaneously with S19 and GST-GFP, generated anti-GFP antibodies early after vaccination. However, after this immunization protocol, antibodies against GFP decreased sharply. In contrast, mice inoculated simultaneously with S19-GFP and GST-GFP induced anti-GFP antibodies for a protracted period. Altogether this suggest that the constitutive expression of GFP by the S19-GFP is necessary to maintain an adequate level of anti-GFP antibodies in mice. Finally, S19-GFP induced in mice a protective efficacy similar to that provided by the corresponding parental S19 strain (Table 2).

### Stability of S19-GFP vaccine and GST-GFP

We have previously shown that the recombinant GFP used for this study is highly stable to high temperature and UV radiation, and that the endotoxic activity of the purified GFP and GST-GFP were practically null (< 0.015 EU/mL) [37]. The stability of the *gfp* gene inserted in chromosome II was assessed by PCR in bacteria isolated from the organs of infected mice and after 20 subcultures in BAB plates. Three CFUs recovered and tested at different time points had the mini-*Tn7*-GFP genotype and were fluorescent, demonstrating the stability of the mini-*Tn7* insertion.

### S19-GFP vaccination in cattle induces anti-GFP antibodies

**Fig 3:**
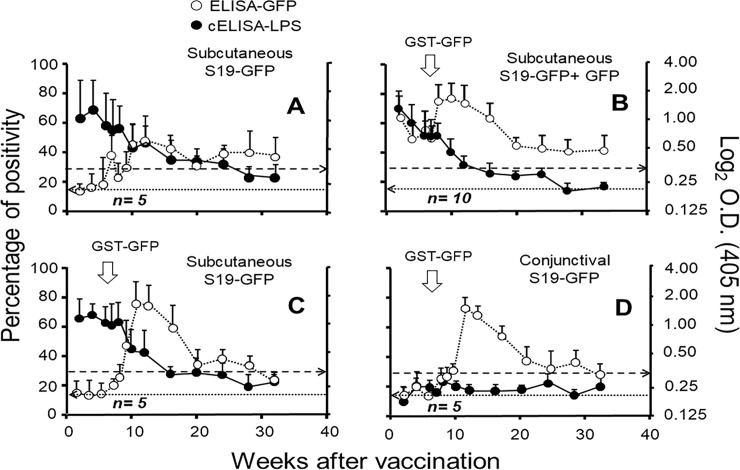
Antibody responses against S-LPS and GFP in heifers vaccinated with S19-GFP following different immunization protocols. Twenty-five heifers were divided into four groups (A-D) (Table 1). Except for experimental group “A”, all other groups were boosted with 150 μg of GST-GFP in calcium alginate after seven weeks of vaccination (white arrows). OD kinetics for each vaccinated group was followed for 32 weeks by cELISA-S-LPS (expressed as % of positivity) and iELISA-GFP expressed as log_2_ OD). The broken horizontal line from left to right marks the cut-off level for the cELISA-LPS. The dotted horizontal line from right to left indicates the ELISA-GFP OD cut-off.

Except for animals of the non-boosted group A (Fig 3A and Table 1), the other groups boosted with GST-GFP produced a quick anti-GFP antibody response (Fig 3B–3D). These results indicate an anamnestic response against GFP in the vaccinated boosted animals, which was independent of the vaccination route. As expected, animals simultaneously immunized with S19-GFP and GFP induced higher iELISA-GFP OD values during the first weeks after vaccination (Fig 3B). The only exception was heifer 1223 (from group B), which had negative or low positive OD values. This animal was RBT negative at week twenty-four after vaccination (S1 Table). Likewise, heifer 95A (from group A) was RBT positive beyond week 64 after vaccination and became RBT negative after 28 months. Nevertheless, this animal remained iELISA-GFP and WB-GFP positive beyond that period (S1 Fig and S1 Table). Thirty-two percent of vaccinated heifers remained iELISA-GFP positive after 28 months.

Most of group D heifers (vaccinated conjunctively) resulted RBT positive but showed low cELISA-S-LPS OD readings, being negative in both tests eight weeks after vaccination (Fig 3 and S1 Table). Nevertheless, most of these group D heifers remained iELISA-GFP positive beyond week 20 after vaccination (S1 Table). Boosting with GST-GFP did not affect the immune response against *Brucella* S-LPS throughout the experiment (Fig 3). Independently of the immunization protocol, all vaccinated heifers resulted WB positive after 24 weeks of vaccination (S1 Fig). It is worth mentioning that those animals that were negative in iELISA-GFP after 24 weeks also resulted negative in RBT (S1 Table). Still, they were positive in WB (S1 Fig).

**Fig 4:**
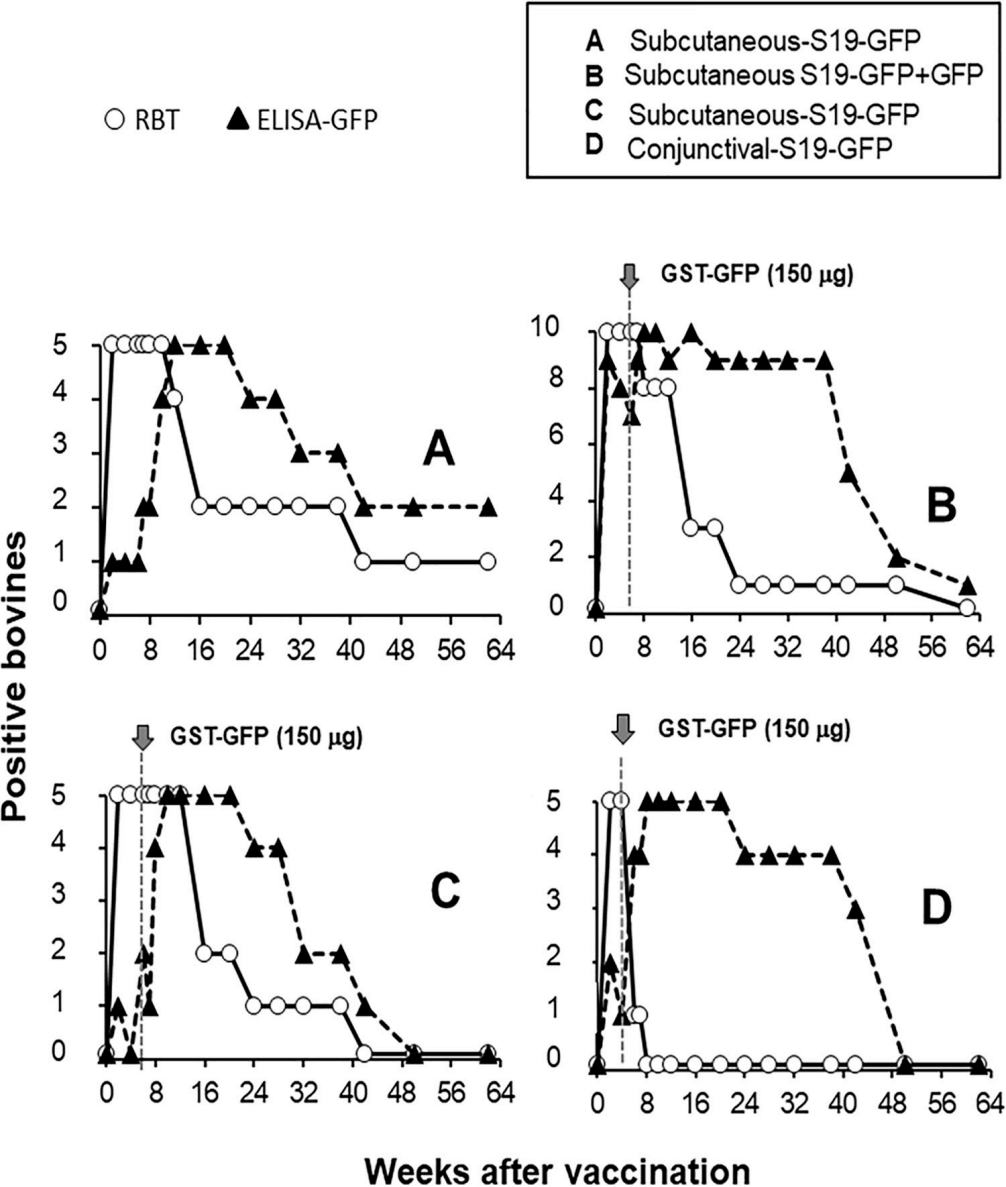
Evolution of the proportion of RBT and iELISA-GFP reactors after different S19-GFP immunization protocols. Twenty-five brucellosis-free heifers were divided into four (A-D) experimental groups (Table 1) and studied serologically for 64 weeks. Except group A, all heifers were boosted with the indicated concentrations of GFP at the specified times (grey arrows).

The evolution of the antibody responses in the iELISA-GFP and the RBT is shown in Fig 4. As expected, the RBT was positive in all animals by the second week after vaccination and lasted for a protracted period; after that, the number of RBT positive animals started to decline (Fig 4A–4D). Except for the subgroup vaccinated with a mixture of S19-GFP and GFP (Fig 4B), all animals from the other groups (including those non-boosted) displayed positive anti-GFP responses longer than the RBT positive reactions (Fig 4A, 4C and 4D).

**Fig 5:**
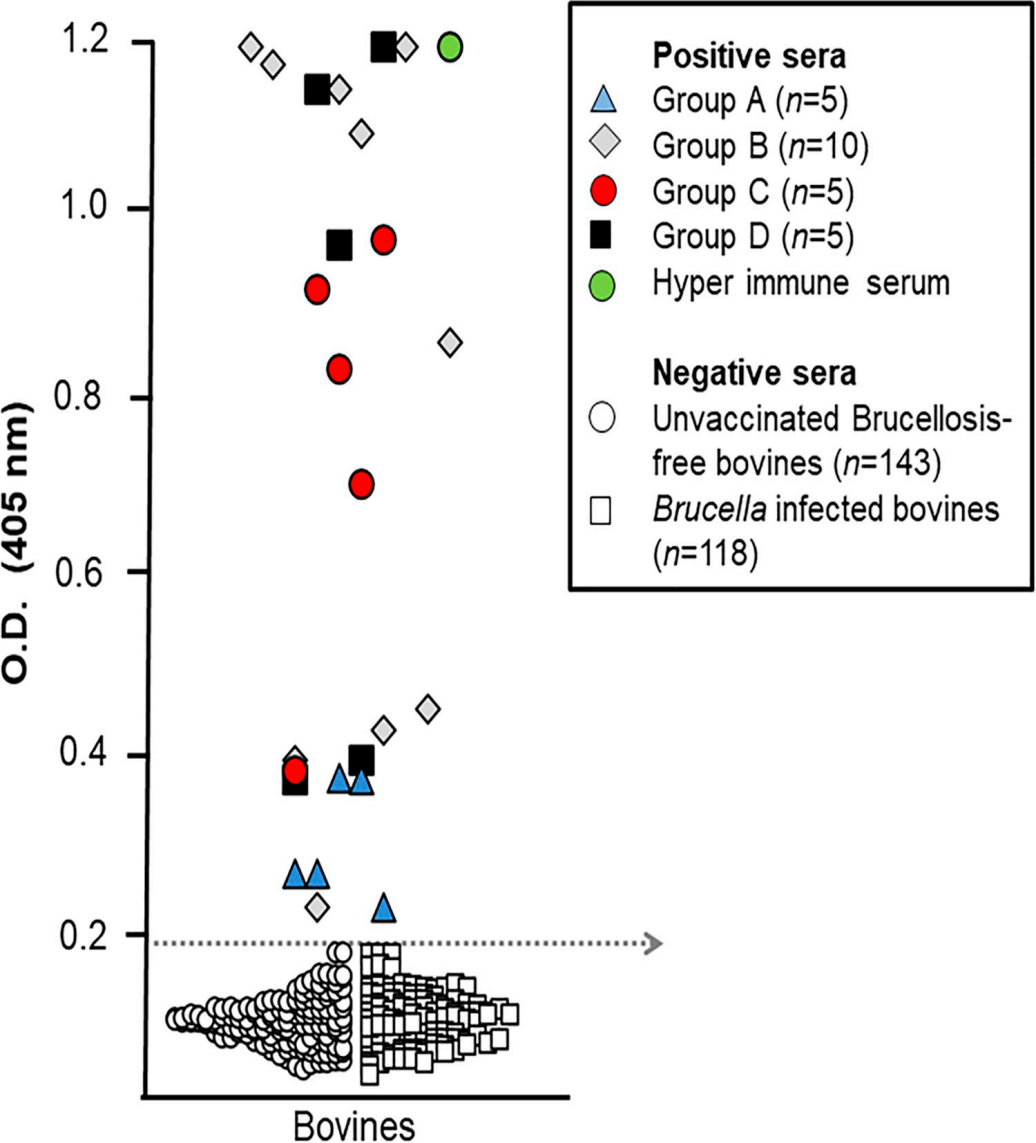
iELISA-GFP validation. The sera of the 25 S19-GFP vaccinated heifers (Table 1) were taken at 16 weeks after vaccination and used as positive controls. The negative control sera (*n* = 143) were taken from brucellosis-free unvaccinated controls (*n* = 118) and sera from all experimental heifers before vaccination (*n* = 25) and 118 *Brucella* infected bovines. The dotted arrow indicates the iELISA-GFP cut-off resulting in 100% diagnostic sensitivity and specificity was estimated as OD = 0.198.

No overlapping was observed in iELISA-GFP OD values obtained between the sera from S19-GFP vaccinated heifers by 16 weeks after vaccination, and those obtained with the 143 unvaccinated brucellosis-free and 118 *Brucella* infected bovine controls, indicating a high specificity (Fig 5). Although the five non-boosted heifers (group A) displayed low iELISA-GFP OD readings at this time, these were above the established cut-off value (Fig 5). Therefore, they were recorded as GFP positives at a given time (S1 Table). Moreover, these non-boosted bovines were also positive against GFP in WB by 24 weeks after vaccination (S1 Fig).

## Discussion

The generation of new *Brucella* vaccines capable of abrogating the diagnostic interferences generated by vaccination has been a recurrent concern throughout the years [22–25, 27, 31–33]. Since most infected cows react against *Brucella* surface antigens, efforts to generate tagged vaccines have concentrated on the removal of relevant surface epitopes (proteins or O-chain and NH) in live-attenuated *Brucella* strains. Though, experience has demonstrated that deleting some relevant antigens from the *Brucella* surface is not straightforward and has several disadvantages. The most obvious drawback is the attenuation beyond the required level of residual virulence, which is critical for vaccine efficacy. Besides, not all infected animals with field virulent *Brucella* strains develop antibodies against the negative epitope selected, leading to a lack of diagnostic sensitivity of the associated diagnostic tests. This characteristic is a recurrent problem in protein-deleted candidates [31, 32, 34, 35]. Finally, vaccination with attenuated *Brucella* strains devoid of relevant molecules and antigens may provide a selective advantage to the fully equipped field virulent brucellae, favoring the potential selection of more pathogenic strains [4].

The generation of bacterial recombinant vaccine candidates for the induction of immune response against foreign antigens is an attractive strategy [47, 48]. The logic behind this is that the infection process works as an adjuvant against the xenogenic protein antigen. Our previous work on *B*. *melitensis* Rev1::*gfp* vaccine in sheep showed promising results for identifying vaccinated animals [37].

Considering these experiences, we developed the *B*. *abortus* S19-GFP vaccine candidate. With the sole exception of the insertion of a stable gene coding for GFP in chromosome II, this candidate displays the same genetic background and shows similar microbiological and biological properties to the parental S19 strain. We demonstrated that immunization of mice with a mixture of S19-GFP and GST-GFP induces a higher and steady antibody response against GFP than the co-administration of the parental S19 and GST-GFP. It seems, therefore, that the intrinsic constitutive expression of GFP by S19-GFP is required to induce an adequate level of anti-GFP antibodies (Fig 2D) as it was demonstrated in the *B*. *melitensis* Rev1::*gfp* vaccine candidate [37].

The S19-GFP subcutaneously immunized heifers displayed a typical cELISA antibody response against *Brucella* S-LPS [49]. Likewise, the cELISA profiles and the number of RBT positives were significantly lower in the animals (group D) vaccinated by the conjunctival route, confirming the low diagnostic interference generated by this vaccination procedure [10, 20, 50]. Although most animals became RBT negative at 32 weeks of vaccination, a few remained RBT positive beyond this period and even one being positive until week 64. Interestingly, this persistent reactor resulted positive in the iELISA-GFP. These animals with persistent anti-S/LPS antibodies are the main source of diagnostic problems in test and slaughter-based eradication programs [10, 15, 16].

Immunization with a single dose of 19-GFP is the most practical protocol according the classical S19 vaccination schemes [10, 51], and thus, boosting with GFP may be impractical. Although the S19-GFP vaccinated but non-GFP boosted heifers (group A) displayed detectable anti-GFP antibodies, the levels were significantly lower than those of the groups boosted with GFP. We do not know if the lower anti-GFP antibody response observed in the non-boosted heifers was due to the relatively mild expression of GFP in the S19-GFP or to intrinsic antigenic properties of the GFP, and this would be investigated. An obvious alternative would be to increase the level of GFP expression in the GFP-S19 vaccine by inserting more copies of the *gfp* gene or expressing GFP on the outer membrane. The challenge would be to perform these changes without affecting the biological and immunological properties of the S19 vaccine background.

The fact that GFP is absent in mammalian hosts or their commensal microorganisms [52], reduces the possibilities of cross-reactions in serological tests. Likewise, the fluorescent phenotype of the *Brucella*-GFP vaccines allows their straightforward recognition by simple visualization techniques, and by a multiplex PCR. Although the production and testing of attenuated *Brucella* vaccines are not problem-free, the accumulated experience already obtained with the parental S19 vaccine [10] should help in performing broader testing of the S19-GFP vaccine candidate.

## Conclusions

We showed that an S19 construct constitutively expressing GFP (S19-GFP) maintains the biological and immunological properties as the parental S19 reference vaccine strain. GFP constitutive expression in S19 is required to maintain an anti-GFP response in animals. S19-GFP induced anti-GFP antibodies in vaccinated cows, reinforcing the principle that S19-GFP vaccine may serve for the identification of S19 vaccinated in brucellosis infected contexts. Further research is required to improve the GFP immunogenicity of the S19-GFP prototype to administrate as a single vaccine dose without GFP boosting.

## Supporting information

S1 Fig WB against GFP of sera from heifers vaccinated with S19-GFP. After 24 weeks of vaccination with S19-GFP, sera of the 25 heifers were tested against purified GFP in WB. All S19-GFP immunized animals demonstrated positive reaction against GFP. Under the conditions tested, none of the sera of pre-immune bovines showed positive reactions in WB. Positive control from GFP hyperimmunized bovine (C+) and negative bovine (C-) control sera, are shown in the figure.
(TIF)

S1 Table Serological results of heifers immunized with S19-GFP. (PDF)

S1 Raw images Raw images of gels and blots. (PDF)

S1 File. The ARRIVE guidelines checklist. (PDF)
