# Supplementary figures and images for: Brucella abortus S19 GFP-tagged vaccine allows the serological identification of vaccinated cattle
Source: PLoS One. 2021 Nov 22;16(11):e0260288. doi: 10.1371/journal.pone.0260288 (PMC8608319; doi:10.1371/journal.pone.0260288)

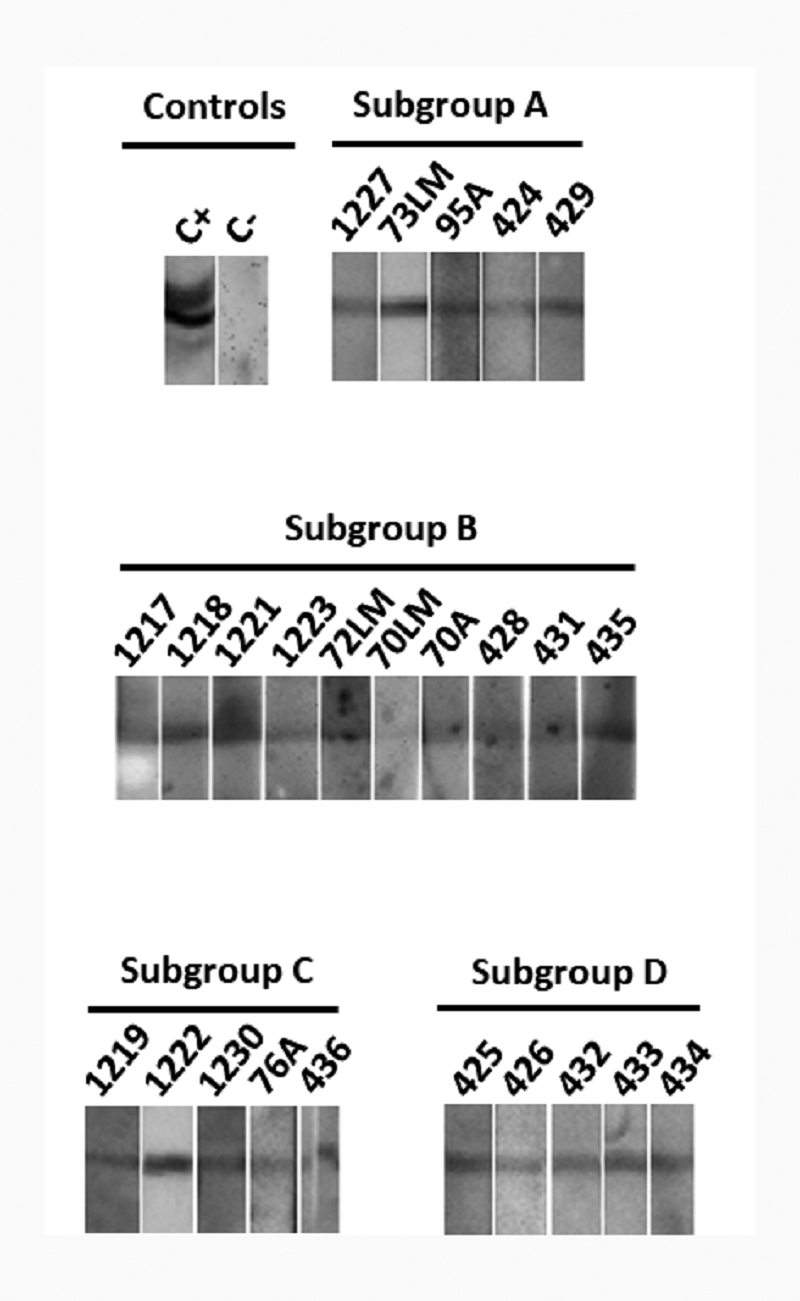

Supplement: S1 Fig — After 24 weeks of vaccination with S19-GFP, sera of the 25 heifers were tested against purified GFP in WB. All S19-GFP immunized animals demonstrated positive reaction against GFP. Under the conditions tested, none of the sera of pre-immune bovines showed positive reactions in WB. Positive control from GFP hyperimmunized bovine (C+) and negative bovine (C-) control sera, are shown in the figure. (TIF) [file pone.0260288.s001.tif]
