# Supplementary material for: Brucella abortus S19 GFP-tagged vaccine allows the serological identification of vaccinated cattle
Source: PLoS One. 2021 Nov 22;16(11):e0260288. doi: 10.1371/journal.pone.0260288 (PMC8608319; doi:10.1371/journal.pone.0260288)
Supplement: S1 Table — (PDF) [file pone.0260288.s002.pdf]

Supplementary Table S1. Serological results of heifers immunized with S19-GFP

Vaccination protocol	Heifer	Assay	Weeks after vaccination																	
			**0**	**2**	**4**	**6**	**7**	**8**	**10**	**12**	**16**	**20**	**24**	**28**	**32**	**38**	**42**	**50**	**64**	**130**
**Group A** Subcutaneous S19-GFP	1227	RBT	-	+	+	+	+	+	+	+	-	-	-	-	-	-	-	-	-	-
		ELISA-GFP	-	-	-	-	+	-	+	+	+	+	+*	+	-	-	-	-	-	-
	73LM	RBT	-	+	+	+	+	+	+	-	-	-	-	-	-	-	-	-	-	ND
		ELISA-GFP	-	-	-	-	-	-	+	+	+	+	-*	-	-	-	-	-	-	ND
	95A	RBT	-	+	+	+	+	+	+	+	+	+	+	+	+	+	+	+	+	-
		ELISA-GFP	-	-	-	-	-	-	-	+	+	+	+*	+	+	+	+	+	+	+*
	424	RBT	-	+	+	+	+	+	+	+	+	+	+	+	+	+	-	-	-	-
		ELISA-GFP	-	-	-	-	-	+	+	+	+	+	+*	+	+	+	+	+	+	-
	429	RBT	-	+	+	+	+	+	+	+	-	-	-	-	-	-	-	-	-	-
		ELISA-GFP	-	+	+	+	+	+	+	+	+	+	+*	+	+	+	-	-	-	-
**Group B** Subcutaneous S19-GFP/GFP 7 weeks after GFP boost	1217	RBT	-	+	+	+	+	+	+	+	-	-	-	-	-	-	-	-	-	-
		ELISA-GFP	-	+	+	+	+	+	+	+	+	+	+*	+	+	+	-	-	-	+*
	1218	RBT	-	+	+	+	+	+	+	+	-	-	-	-	-	-	-	-	-	-
		ELISA-GFP	-	+	+	+	+	+	+	+	+	+	+*	+	+	+	+	-	-	+
	1221	RBT	-	+	+	+	+	+	+	+	+	+	-	-	-	-	-	-	-	-
		ELISA-GFP	-	+	+	-	+	+	+	+	+	+	+*	+	+	+	+	-	-	-
	1223	RBT	-	+	+	+	+	+	+	+	+	+	-	-	-	-	-	-	-	-
		ELISA-GFP	-	-	-	-	-	+	+	-	+	-	-*	-	-	-	-	-	-	-
	70LM	RBT	-	+	+	+	+	+	+	+	+	+	+	+	+	+	+	+	-	-
		ELISA-GFP	-	+	+	+	+	+	+	+	+	+	+*	+	+	+	+	+	+	-
	72LM	RBT	-	+	+	+	+	+	+	+	-	-	-	-	-	-	-	-	-	-
		ELISA-GFP	-	+	+	+	+	+	+	+	+	+	+*	+	+	+	+	-	-	+*
	70A	RBT	-	+	+	+	+	-	-	-	-	-	-	-	-	-	-	-	-	-
		ELISA-GFP	-	+	-	+	+	+	+	+	+	+	+*	+	+	+	-	-	-	-
	428	RBT	-	+	+	+	+	-	-	-	-	-	-	-	-	-	-	-	-	-
		ELISA-GFP	-	+	+	+	+	+	+	+	+	+	+*	+	+	+	-	-	-	+*

	431	RBT	-	+	+	+	+	+	+	+	-	-	-	-	-	-	-	-	-	-
		ELISA-GFP	-	+	+	+	+	+	+	+	+	+	+*	+	+	+	+	+	-	-
	435	RBT	-	+	+	+	+	+	+	+	-	-	-	-	-	-	-	-	-	-
		ELISA-GFP	-	+	+	-	+	+	+	+	+	+	+*	+	+	+	-	-	-	+*
**Group C** Subcutaneous S19-GFP 7 weeks after GFP boost	1219	RBT	-	+	+	+	+	+	+	+	-	-	-	-	-	-	-	-	-	-
		ELISA-GFP	-	-	-	-	-	+	+	+	+	+	+*	+	-	-	-	-	-	-
	1222	RBT	-	+	+	+	+	+	+	+	-	-	-	-	-	-	-	-	-	-
		ELISA-GFP	-	-	-	+	-	+	+	+	+	+	+*	+	+	+	-	-	-	-
	1230	RBT	-	+	+	+	+	+	+	+	+	+	+	+	+	+	-	-	-	-
		ELISA-GFP	-	+	-	+	+	+	+	+	+	+	+*	+	+	+	+	-	-	-
	76A	RBT	-	+	+	+	+	+	+	+	-	-	-	-	-	-	-	-	-	-
		ELISA-GFP	-	-	-	-	-	-	+	+	+	+	+*	+	-	-	-	-	-	-
	436	RBT	-	+	+	+	+	+	+	+	+	+	-	-	-	-	-	-	-	-
		ELISA-GFP	-	-	-	-	-	+	+	+	+	+	-*	-	-	-	-	-	-	+
**Group D** Conjunctival S19-GFP 7 weeks after GFP boost	425	RBT	-	+	+	-	-	-	-	-	-	-	-	-	-	-	-	-	-	-
		ELISA-GFP	-	+	+	+	+	+	+	+	+	+	+*	+	+	+	+	-	-	+*
	426	RBT	-	+	+	-	-	-	-	-	-	-	-	-	-	-	-	-	-	-
		ELISA-GFP	-	-	-	-	-	+	+	+	+	+	+*	+	+	+	-	-	-	-
	432	RBT	-	+	+	-	-	-	-	-	-	-	-	-	-	-	-	-	-	-
		ELISA-GFP	-	+	-	+	+	+	+	+	+	+	+*	+	+	+	+	-	-	-
	433	RBT	-	+	+	-	-	-	-	-	-	-	-	-	-	-	-	-	-	-
		ELISA-GFP	-	-	-	+	+	+	+	+	+	+	+*	+	+	+	+	-	-	-
	434	RBT	-	+	+	+	+	-	-	-	-	-	-	-	-	-	-	-	-	-
		ELISA-GFP	-	-	-	+	+	+	+	+	+	+	-*	-	-	-	-	-	-	-

Results are RBT and iELISA-GFP, exception for week 130, which were tested also by WB for GFP. (-) Negative reaction; (+) Positive reaction. (*) Indicates positive reaction in WB for GFP, (ND) not determined.
