# Supplementary material for: Brucella abortus S19 GFP-tagged vaccine allows the serological identification of vaccinated cattle
Source: PLoS One. 2021 Nov 22;16(11):e0260288. doi: 10.1371/journal.pone.0260288 (PMC8608319; doi:10.1371/journal.pone.0260288)
Supplement: S1 Raw images — (PDF) [file pone.0260288.s003.pdf]

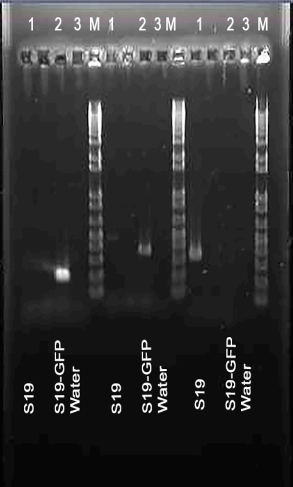

Figure 1B.
UVP Photo Doc-It Imaging System

M: Lane with DNA ladder

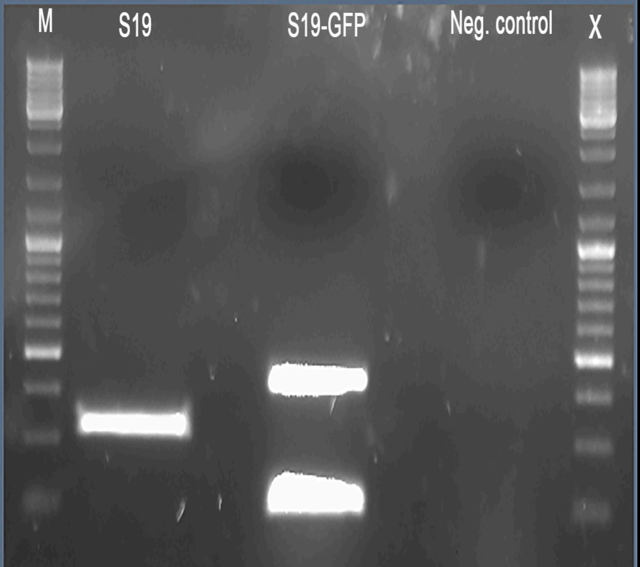

Figure 1D. UVP Photo Doc-IT imaging System
M: Lane with DNA ladder

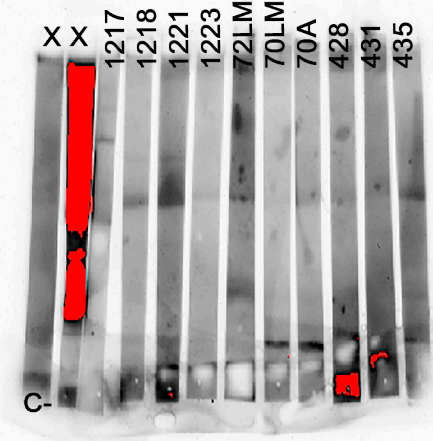

Supplemental Figure 1.Subgroup B
ChemiDoc Imaging Systems
Membrane contains purified GFP
Each strip sera from different cows

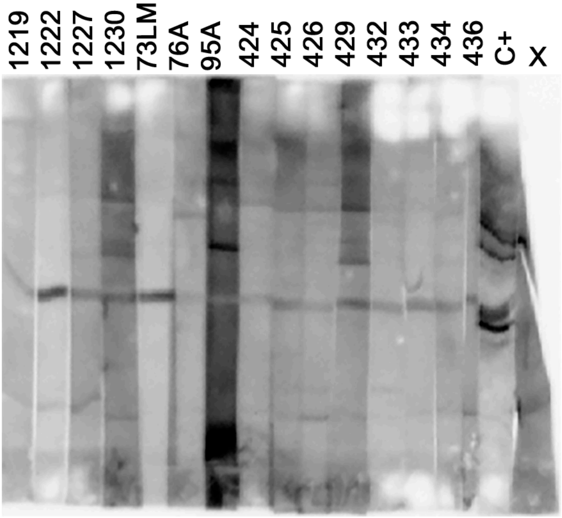

Supplemental Figure 1.Subgroup A,C,D
ChemiDoc Imaging Systems
Membrane contains purified GFP
Each strip sera from different cows
